# Supplementary material for: Multiple proteases are involved in mesothelin shedding by cancer cells
Source: Commun Biol. 2020 Dec 1;3:728. doi: 10.1038/s42003-020-01464-5 (PMC7708464; doi:10.1038/s42003-020-01464-5)
Supplement: Supplementary file 1 — Supplementary Information [file 42003_2020_1464_MOESM1_ESM.pdf]

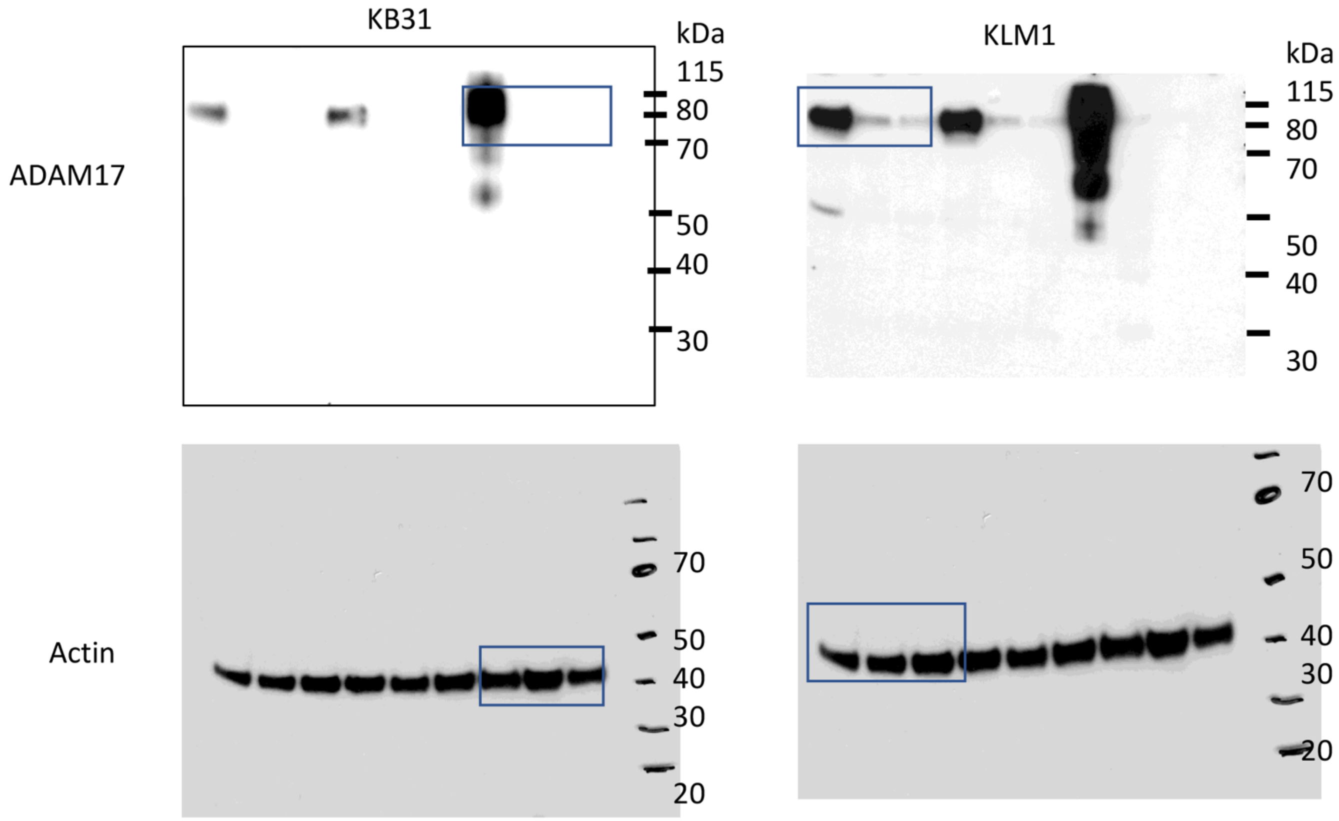

Supplemental Figure 1: Full-sized scans of Immunoblots in Figure 2a

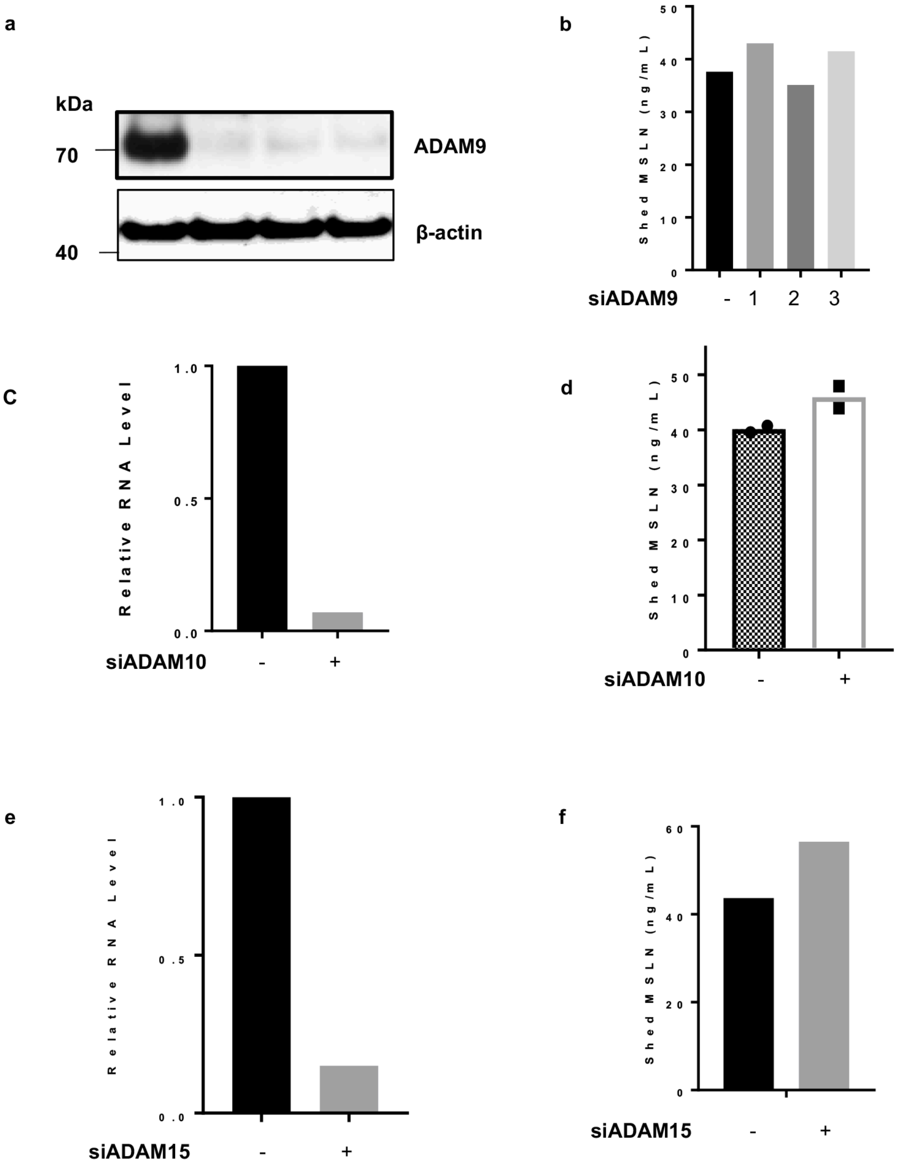

Supplementary Figure 2. Knock down of ADAMs did not affect MSLN shedding in KB31 cells. a, b, 3 different siRNA oligos for ADAM9 were transfected into KB31 cells. After 72 hr, the protein lysates were analyzed by western blot using anti-ADAM9 antibody **(a,** full image in Supplemental Fig. 12, 2a**)**, and the cell culture media were collected and shed MSLN was measured **(b, n=1)**. **c ,d, e, f,** ADAM10 siRNA **(c, d)** or ADAM15 siRNA **(e, f)** was transfected into cells and after 48 hr, the RNA were analyzed by real time PCR **(c, e)**, and shed MSLN measured in media 48-72 hr post-transfection **(d, n=2, f, n=1)**.

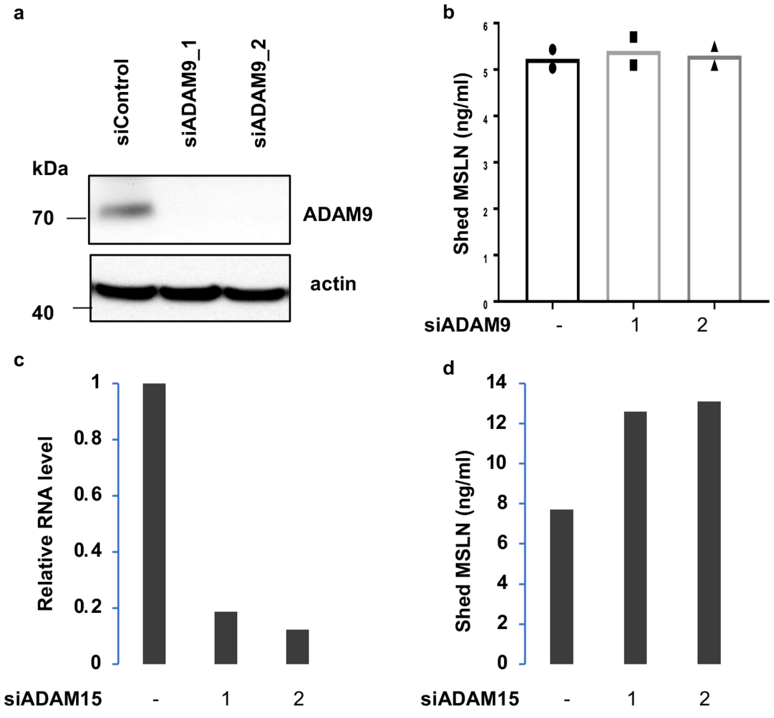

Supplementary Figure 3. Knock down ADAM 9 and ADAM 15 in KLM1 cells did not decrease MSLN shedding. a, b, 2 different siRNA from ADAM9 were transfected into KLM1 cells. After 72 hr, the protein lysates were analyzed by western blot using anti-ADAM9 antibody **(a**, full image in Supplemental Fig. 12, 3a**)**, and the cell culture media were collected and shed MSLN were measured **(b, n=2)**. **c, d,** iADAM15 was transfected, after 48 hr, the RNA was analyzed by real time PCR **(c)** and shed MSLN measured from culture media 48-72 hr post-transfection **(d, n=1)**.

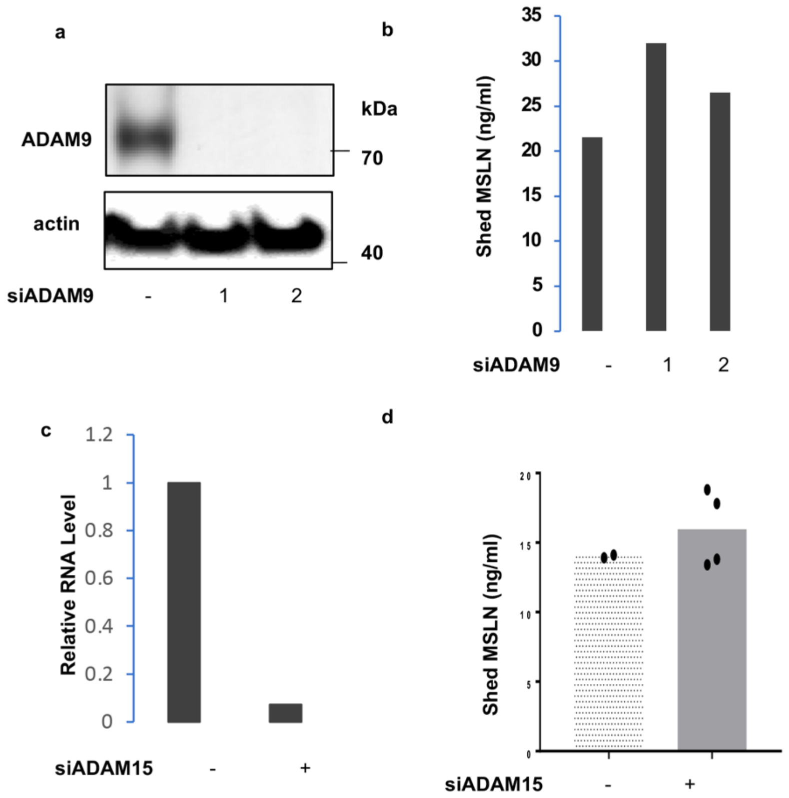

Supplementary Figure 4. Knock down ADAM 9 and ADAM15 did not affect MSLN shedding in OVCAR8 cells. a, b, 2 siRNA oligos for ADAM 9 were transfected into OVCAR 8 cells. After 72 hr of transfection, total protein lysates were analyzed by western blot using anti-ADAM9 Ab. Actin is the loading control **(a**, full image in Supplemental Fig. 12, 4a**)**. Shed MSLN levels in culture media 48-72 hr after transfection **(b, n=1)**. **c, d,** siRNA from ADAM15 were transfected, after 48 hr, the RNA was analyzed by real time PCR using specific ADAM15 primers, actin is the internal control to normalized relative expression **(c)**. Shed MSLN was measured in media 48-72 hr post transfection **(d, n=2 and 4)**.

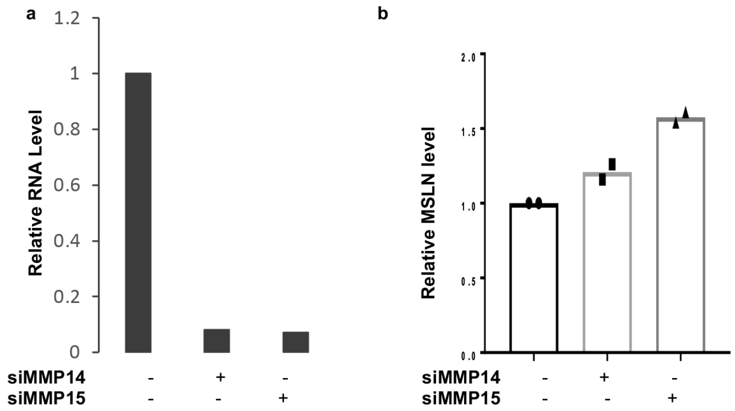

Supplementary Figure 5. Knock down of MMP14 or MMP15 did not lower MSLN shedding in OVCAR8 cells. a, b, MMP14 or MMP15 siRNA was transfected and after 48 hr, MMP14 or MMP15 RNA was analyzed by real time PCR **(a)**; shed MSLN was analyzed 48-72 hr post transfection **(b, n=2)**.

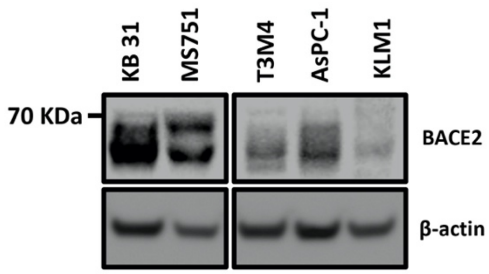

Supplementary Figure 6. Endogenous BACE2 protein expression in 5 cancer cell lines. Western blot of cancer cell lysates show expression of BACE2 in KB31 and MS751 cervical cancer cells, and T3M4, AsPC-1 and KLM1 pancreatic cancer cells. Full image shown in Supplemental Fig. 13.

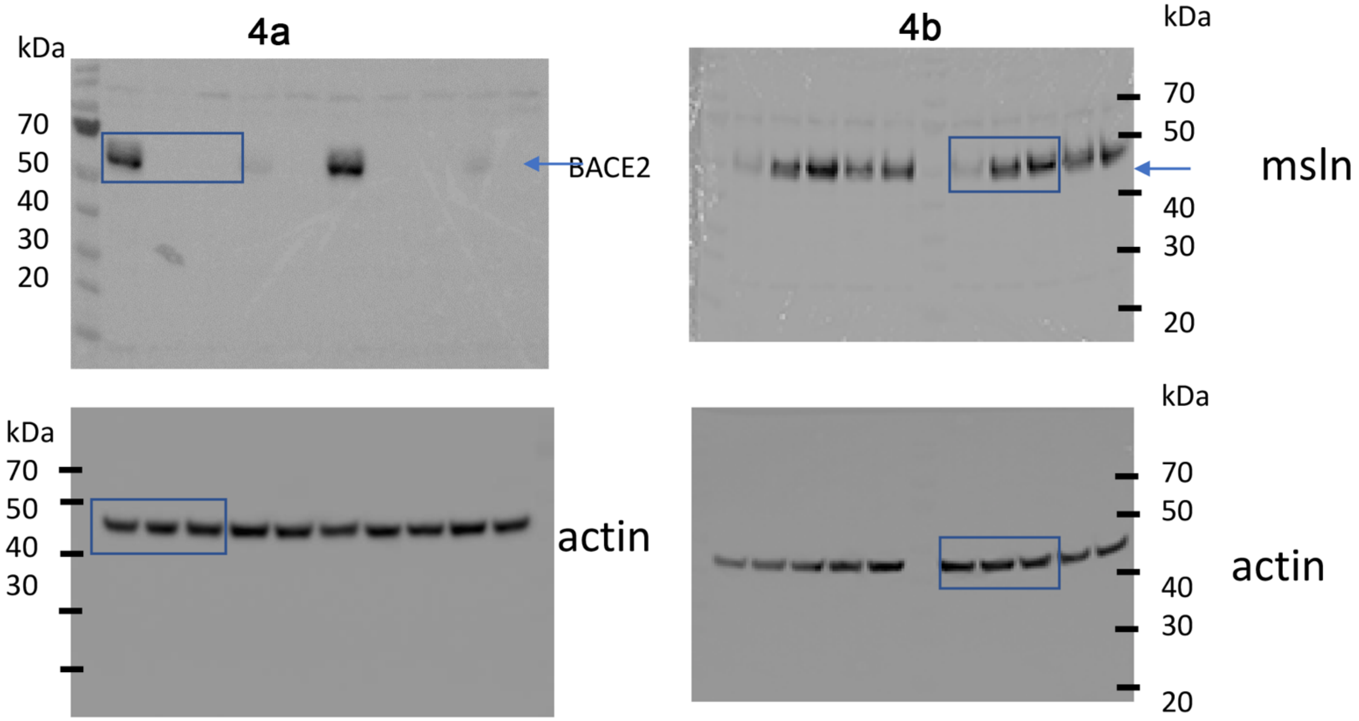

Supplemental Figure 7: Full-sized scans of Immunoblots in Figures 4a and 4b

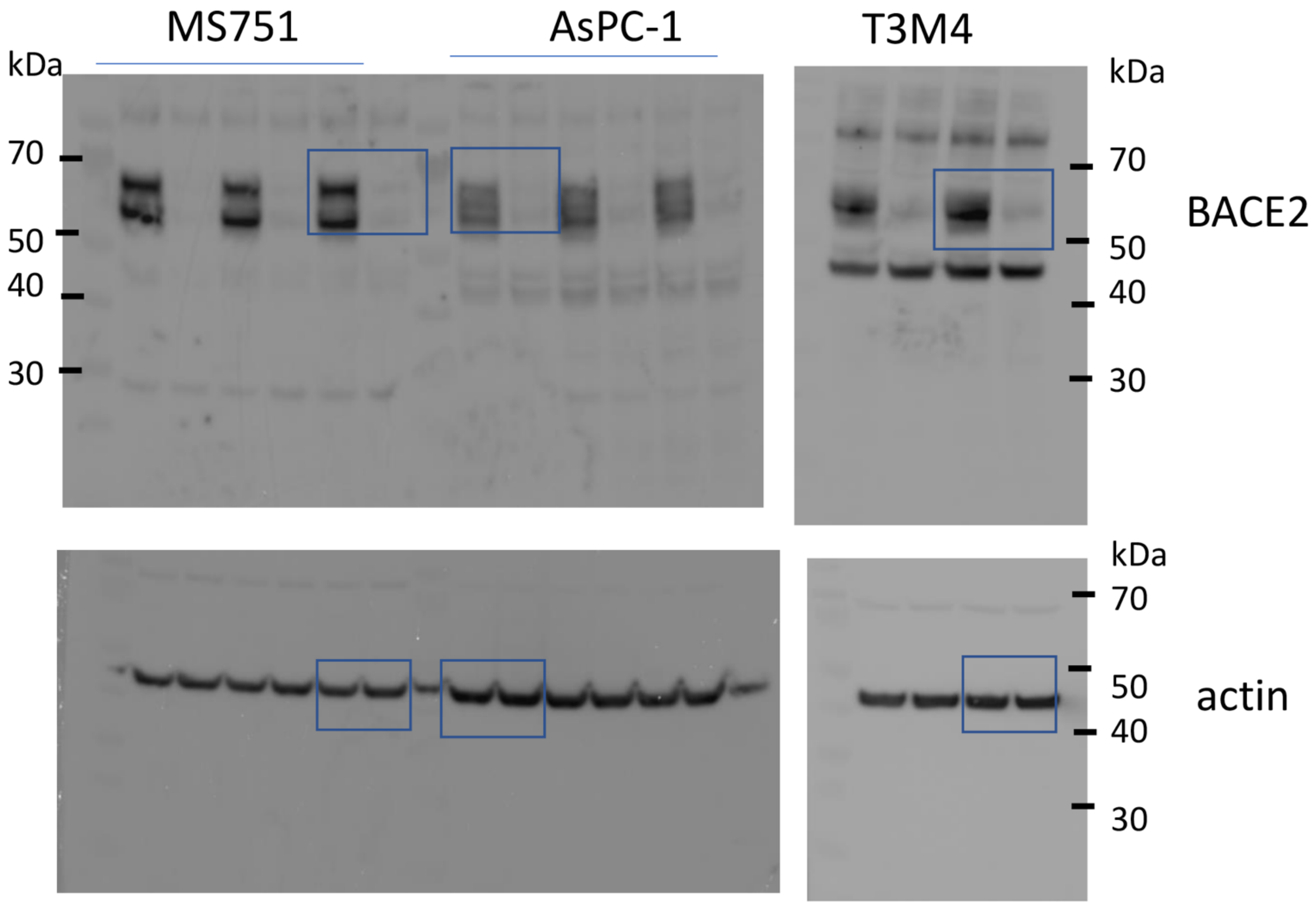

Supplemental Figure 8: Full-sized scans of Immunoblots in Figure 5a

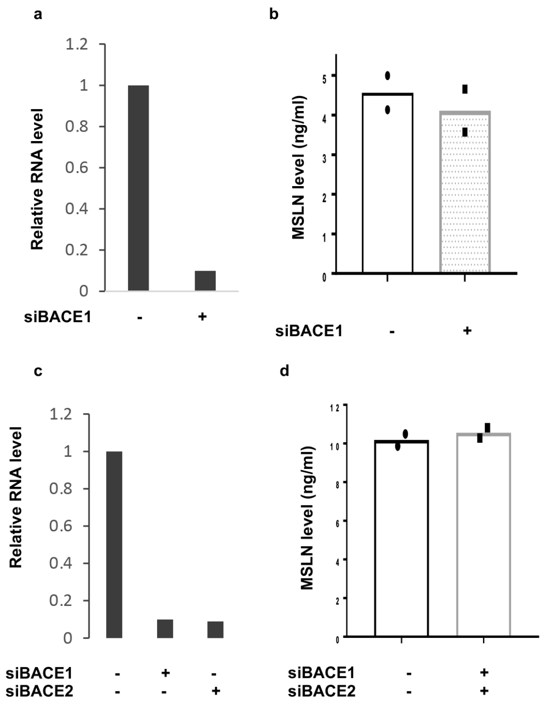

Supplementary Figure 9. Knock down of BACE in KLM1 or OVCAR8 did not affect MSLN shedding.

Real time PCR analysis of siBACE1 (KLM1, **a**) and both BACE1 and BACE2 in OVCAR8 **(c)** 48hr after siRNA treatment. Shed MSLN in KLM1 **(b, n=2)** or OVCAR8 **(d, n=2)** 48-72 hr after transfection.

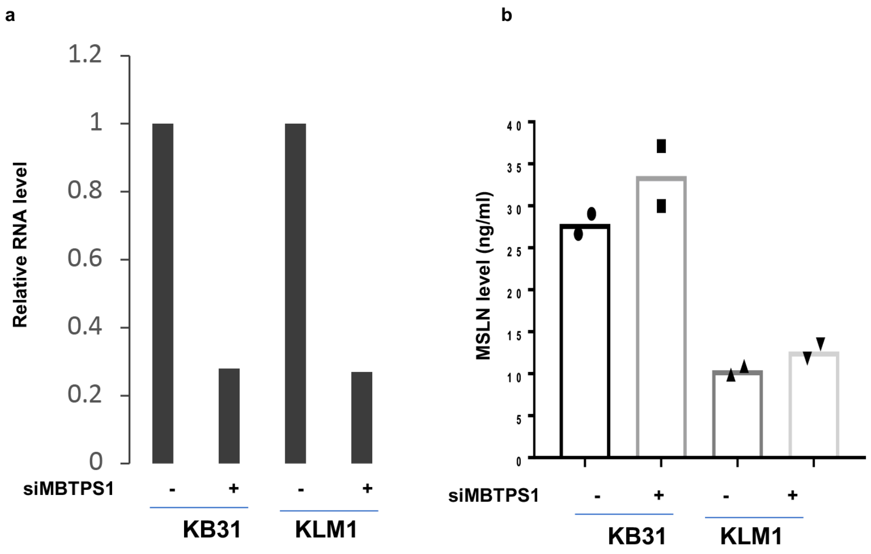

Supplementary Figure 10. Knock down of MBTPS1 does not affect MSLN shedding. siRNA of MBTBS1 was transfected into KB31 or KLM1 cells. RNA levels were analyzed 48 hr later **(a)** and shed MSLN from culture media collected at 48-72 hr was measured **(b, n=2)**.

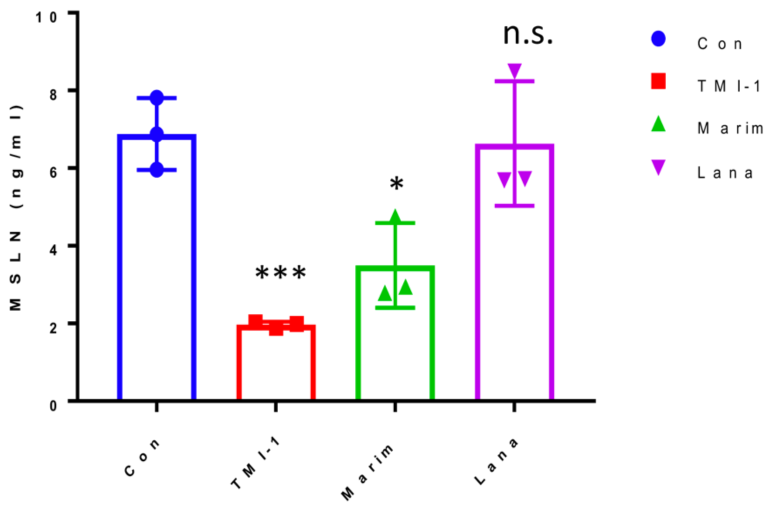

Supplementary Figure 11: MSLN shedding is lowered by ADAM and MMP inhibitors but not BACE inhibitor in RH16 cells. 15,000 RH16 cells were plated in 96 wells. Next morning the cells were washed with culture media 3 times, and 10 µM DMSO (Con), 10 µM TMI-1, 10 µM Marimastat (Marim) or 10 µM Lanabecestat (Lana) were added; 48hr later shed MSLN was measured in culture media. Cell viability was also measured by WST-8 assay when cell media were collected and data corrected for cell growth (P=0.0008, 0.015, 0.826, respectively. n.s.=not significant, *P<0.05, ***P<0.001, n=3)

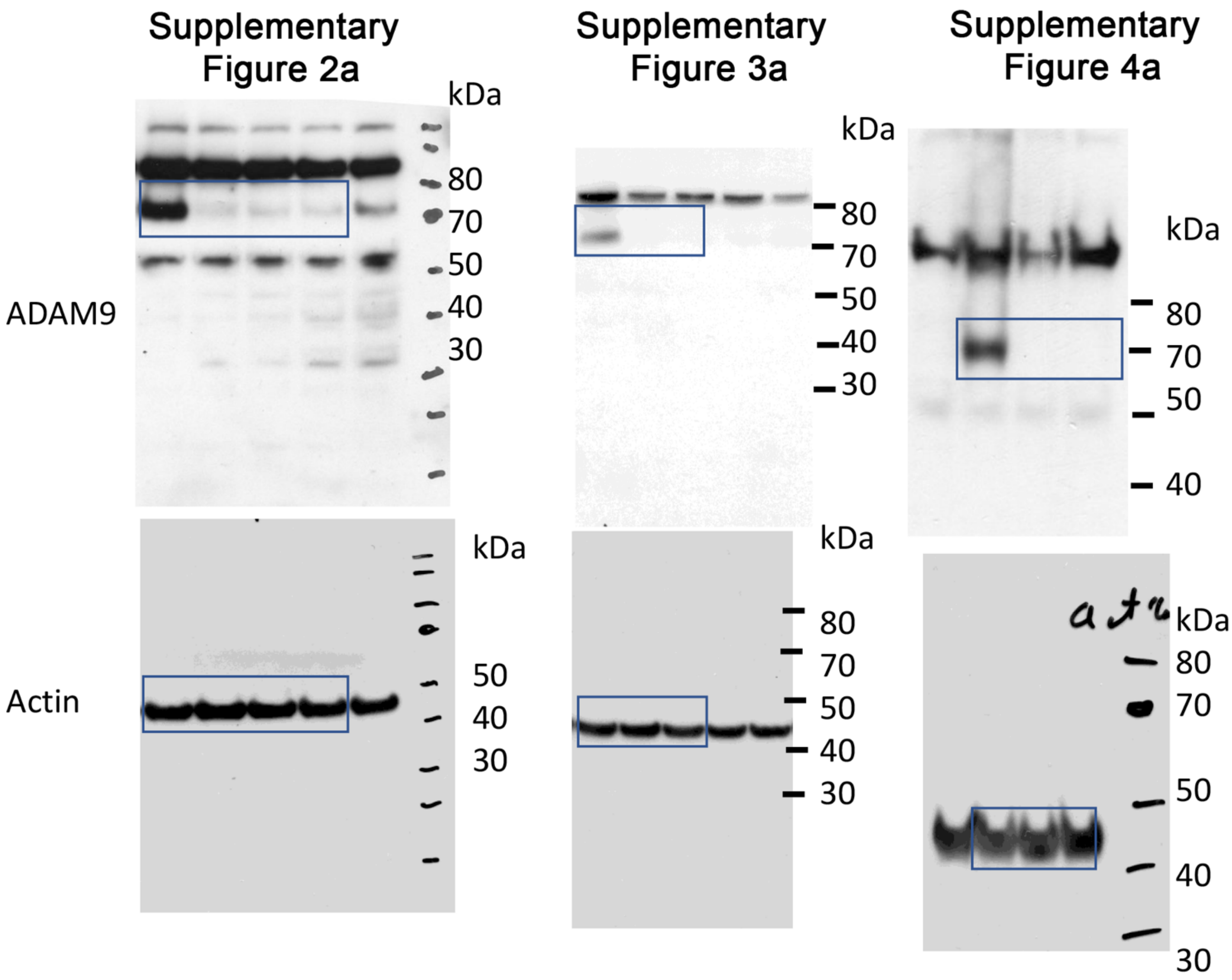

Supplemental Figure 12: Full-sized scans of Immunoblots in Supplemental Figures 2a, 3a and 4a

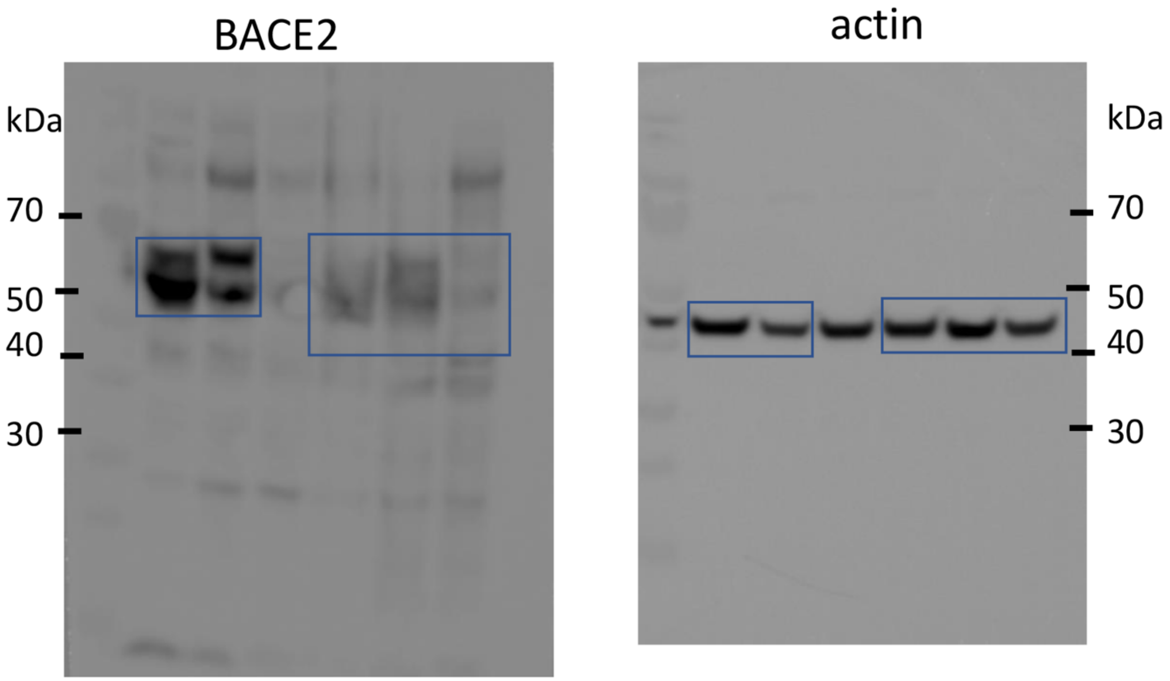

Supplemental Figure 13: Full-sized scans of Immunoblots in Supplemental Figure 6a

Samples	C-terminal sequences
KB31	[R-].QRQDDLDTLGLGLQGGIPNGYLVL.[D]
	[R-].QRQDDLDTLGLGLQGGIPNGYLVLDL.[S]
RH16	[R].QRQDDLDTLGLGLQGGIPNGY[L]
KLM1	[N].GYLVLDLSMQEA.[-]
	[R-].QRQDDLDTLGLGLQGGIPN.[G]
	[R-].QRQDDLDTLGLGLQGGIPNG.[Y]
	[R-].QRQDDLDTLGLGLQGGIPNGY.[L]
	[R-].QRQDDLDTLGLGLQGGIPNGYLVLD.[L]
OVCAR8	[R-].QRQDDLDTLGLGLQGGIPN.[G]
	[R-].QRQDDLDTLGLGLQGGIPNG.[Y]
	[R-].QRQDDLDTLGLGLQGGIPNGY.[L]
	[R-].QRQDDLDTLGLGLQGGIPNGYLVLD.[L]
A431/H9	[R-].QRQDDLDTLGLGLQGGIPN.[G]
	[R-].QRQDDLDTLGLGLQGGIPNGYLVLDL.[S]

Supplementary Table 1: GC-MS identification of C-terminal peptides of MSLN in purified cell culture media.

Cancer	Cell lines	ADAM9	ADAM10	ADAM15	ADAM17	BACE1	BACE2	MMP14	MMP15
Cervical	KB31	**19**	**29**	**87**	**8**	**5**	**20**	0	**9**
Pancreatic	KLM1	**29**	**50**	**34**	**18**	7	0.1	**61**	**21**
Ovarian	OVCAR8	**92**	**20**	**22**	**11**	**2.5**	**5.5**	**32**	**8**
Pancreatic	T3M4	64	29	52	14	6	**6**	65	8
Pancreatic	AsPC1	117	29	27	9	7	**16**	33	16
Cervical	MS751	5	18	70	6	0.5	**14**	54	12
Mesothelioma	RH16	105	48	16	11	21	7	7	4
Epidermoid	A431/H9	132	50	29	35	4	3	55	13

Supplementary Table 2: Expression of ADAMs, Mt-MMPs and BACEs in various cell lines.

Data on cell lines KB31, KLM1 and RH16 was obtained at NCI. A431/H9 is from GEO study GSE131132; all other data is from CCLE. Bold indicates the gene was knocked down. Bold red indicates knock down resulted in decreased MSLN expression and the data is derived from this study. The units are RPKM (Reads per kilo base per million mapped reads).

Cells	20 µM Lana	MSLN (ng/ml)	HER1 (pg/ml)	TGF-β1 (pg/ml)	FOLR1 (pg/ml)	CA125 (pg/ml)	Galectin 3 (pg/ml)	TNF-α (pg/ml)
KB31	No	23.1 ± 2.0	36 ± 1.3	4784 ± 441	33330 ± 933	129 ± 1.4	1568 ± 36	7 ± 0.7
	Yes	12.4 ± 0.8*	24 ± 1.0**	4443 ± 548	34842 ± 1974	135 ± 0.4	1421 ± 59	5 ± 0.35
MS751	No	10.6 + 0.5	842 ± 46	1932 ± 216	20 ± 0.2	61 ± 1.2	858 ± 1.0	1
	Yes	5.6 ± 0.3**	635 ± 35*	1992 ± 44	23 ± 1.7	52 ± 1.4*	864 ± 3.4	1
KLM1	No	8.7 ± 0.3	68 ± 3.7	2004 ± 81	47 ± 2.9	411 ± 22	1447 ± 50	0
	Yes	7.7 ± 0.04	40 ± 3.1*	1920 ± 152	43 ± 1.3	356 ± 18	1119 ± 51	3 ± 0.1
AsPC1	No	17.7 ± 0.06	39 ± 3.7	1454 ± 144	<14.9	<2.7	2790 ± 131	2 ± 0.3
	Yes	14.1 ± 0.02***	24 ± 2.1*	1230 ± 1.4	<14.9	<2.7	2534 ± 30	1 ± 0.2
T3M4	No	28.4 ± 0.26	636 ± 50	2656 ± 12	115 ± 2.9	733 ± 78	366 ± 0.7	3 ± 0.8
	Yes	19.5 ± 0.22***	686 ± 9	2810 ± 125	109 ± 3.0	651.4 ± 55	352 ± 16	3 ± 0.3

Supplementary Table 3: Screen of BACE inhibitors on several shed proteins. 20 µM of BACE inhibitor Lanabecestat (Lana) was added to the cells. After overnight culture, the culture media was collected and ELISAs were performed by AssayGate.

Name	Sequences	Purpose	Sources
siADAM9_1	GAGATTAACTAGAGAAAGA	knock down	Dharmacon
siADAM9_2	GGAGGGAGTTCATAATTCA	knock down	Dharmacon
siADAM9_3	GTGCACAGCTAGTTCTAAA	knock down	Dharmacon
siADAM10	GAATGGTATAACAAGGTGA	knock down	Ambion
siADAM15_1	GATTGTGACTTGCCTGAAT	knock down	Dharmacon
siADAM15_2	GGACCCACCTGCCAGTACA	knock down	Dharmacon
siADAM17_1	GGATGTAATTGAACGATTT	knock down	Ambion
siADAM17_2	GGCGATCACGAGAACAAT	knock down	Ambion
siMMP14_1	GGATGGACACGGAGAATTT	knock down	Dharmacon
siMMP15_1	CCGGTGTGCTCGACGAAGA	knock down	Dharmacon
siMMP15_2	CATCTGACCTTTAGCATCC	knock down	Dharmacon
siBACE1	UAUGGGAGCUGUUAUCAUG	knock down	Dharmacon
siBACE2_1	CAACGGAGGTAGTCTTGTC	knock down	Dharmacon
siBACE2_2	GGGATTAAATGGAATGGAA	knock down	Dharmacon
ADAM9-For	TCCATTGCTCTTAGCGACTGT	Real time	Lofstrand
ADAM9-Rev	GGGGTTCAATCCCATAACTCG	Real time	Lofstrand
ADAM10-For	TTTCAACCTACGAATGAAGAGGG	Real time	Lofstrand
ADAM10-Rev	TAAAATGTGCCACCACGAGTC	Real time	Lofstrand
ADAM15-For	CAGGACGATCTCCCAATTAGC	Real time	Lofstrand
ADAM15-Rev	GGACCAACTCCCTATTCTGTAGC	Real time	Lofstrand
ADAM17-For	GACTCTAGGGTTCTAGCCCAC	Real time	Lofstrand
ADAM17-Rev	GGAGACTGCAAACGTGAAACAT	Real time	Lofstrand
MMP14-For	CGAGGTGCCCTATGCCTAC	Real time	Lofstrand
MMP14-Rev	CTCGGCAGAGTCAAAGTGG	Real time	Lofstrand
MMP15-For	AGGTCCATGCCGAGAACTG	Real time	Lofstrand
MMP15-Rev	GTCTCTTCGTCGAGCACACC	Real time	Lofstrand
BACE1-For	ACCAACCTTCGTTTGCCCAA	Real time	Lofstrand
BACE1-Rev	TCTCCTAGCCAGAAACCATCAG	Real time	Lofstrand
BACE2-For	GGAGATGCTGATCGGGACC	Real time	Lofstrand
BACE2-Rev	AGTACGTGTCTATGTAGGAGTGC	Real time	Lofstrand
ACTIN-For	CCGGCCAGCCAGGTCCAGAC	Real time	Lofstrand
ACTIN-Rev	CCAAGGCCAACCGCGAGAAGAT	Real time	Lofstrand

Supplementary Table 4: Sequence data of oligo nucleotides.
