# Supplementary material for: Multiple proteases are involved in mesothelin shedding by cancer cells
Source: Commun Biol. 2020 Dec 1;3:728. doi: 10.1038/s42003-020-01464-5 (PMC7708464; doi:10.1038/s42003-020-01464-5)
Supplement: Supplementary file 4 — Reporting Summary [file 42003_2020_1464_MOESM4_ESM.pdf]

nature research

Corresponding author(s): Ira pastan

Last updated by author(s): Oct 15, 2020

Reporting Summary

Nature Research wishes to improve the reproducibility of the work that we publish. This form provides structure for consistency and transparency in reporting. For further information on Nature Research policies, seeAuthors & Referees and theEditorial Policy Checklist .

Statistics

For all statistical analyses, confirm that the following items are present in the figure legend, table legend, main text, or Methods section.

n/a	Confirmed	
☐	☒	The exact sample size (*n*) for each experimental group/condition, given as a discrete number and unit of measurement
☒	☐	A statement on whether measurements were taken from distinct samples or whether the same sample was measured repeatedly
☐	☒	The statistical test(s) used AND whether they are one- or two-sided<br>*Only common tests should be described solely by name; describe more complex techniques in the Methods section.*
☒	☐	A description of all covariates tested
☒	☐	A description of any assumptions or corrections, such as tests of normality and adjustment for multiple comparisons
☒	☐	A full description of the statistical parameters including central tendency (e.g. means) or other basic estimates (e.g. regression coefficient) AND variation (e.g. standard deviation) or associated estimates of uncertainty (e.g. confidence intervals)
☒	☐	For null hypothesis testing, the test statistic (e.g. *F*, *t*, *r*) with confidence intervals, effect sizes, degrees of freedom and *P* value noted<br>*Give P values as exact values whenever suitable.*
☒	☐	For Bayesian analysis, information on the choice of priors and Markov chain Monte Carlo settings
☒	☐	For hierarchical and complex designs, identification of the appropriate level for tests and full reporting of outcomes
☒	☐	Estimates of effect sizes (e.g. Cohen's *d*, Pearson's *r*), indicating how they were calculated

Our web collection on statistics for biologists contains articles on many of the points above.

Software and code

Policy information about availability of computer code

Data collection	No software was used.
Data analysis	GraphPad Prism 7 and Microsoft Excel.

For manuscripts utilizing custom algorithms or software that are central to the research but not yet described in published literature, software must be made available to editors/reviewers. We strongly encourage code deposition in a community repository (e.g. GitHub). See the Nature Research guidelines for submitting code & software for further information.

Data

Policy information about availability of data

All manuscripts must include a data availability statement. This statement should provide the following information, where applicable:

- Accession codes, unique identifiers, or web links for publicly available datasets
- A list of figures that have associated raw data
- A description of any restrictions on data availability

Original mass spectra has been deposited in MassIVE (https://massive.ucsd.edu) under the identifier MassIVE: MSV000085950. The source data behind graphs can be found in Source Data. All other data generated during and/or analyzed during the current study are available from the corresponding author on reasonable request.

Field-specific reporting

Please select the one below that is the best fit for your research. If you are not sure, read the appropriate sections before making your selection.

Life sciences study design

All studies must disclose on these points even when the disclosure is negative.

Sample size	Most sample sizes are 3-5 replicates
Data exclusions	no data were excluded in the samples
Replication	Each experiment was repeated 2-3 times.
Randomization	n/a
Blinding	n/a

Reporting for specific materials, systems and methods

We require information from authors about some types of materials, experimental systems and methods used in many studies. Here, indicate whether each material, system or method listed is relevant to your study. If you are not sure if a list item applies to your research, read the appropriate section before selecting a response.

Materials & experimental systems

n/a	Involved in the study	
☐	☒	Antibodies
☐	☒	Eukaryotic cell lines
☒	☐	Palaeontology
☒	☐	Animals and other organisms
☒	☐	Human research participants
☒	☐	Clinical data

Methods

n/a	Involved in the study	
☒	☐	ChIP-seq
☐	☒	Flow cytometry
☒	☐	MRI-based neuroimaging

Antibodies

Antibodies used	Anti-ADAM17 anti-ADAM9 were from Cell Signaling Technology (Cat:3976S and 4151S, both 1:1000); anti-BACE2 antibody was from Santa Cruz Biotechnology (sc-271212, 1:1000); Anti-MSLN antibody (mouse monoclonal MN, 1ug/ml) is from our lab.
Validation	Validation of the antibodies ADAM17, ADAM9 and BACE2 were described in the manufacturers website. Anti-MSLN validation was published in Clin Cancer Res 2005;11:5840-5846.

Eukaryotic cell lines

Policy information about cell lines

Cell line source(s)	KB31, KLM1, AsPC1, A431/H9 cells are from previously published lab stock; OVCAR8 and MS751 cells were purchased from ATCC;T3M4 were from M. Ho (NCI).
Authentication	None of the cell lines were authenticated within 1 year.
Mycoplasma contamination	All cell lines were tested negative for mycoplasma contamination.
Commonly misidentified lines (See ICLAC register)	No misidentified cell lines.

Flow Cytometry

Plots

Confirm that:

☐ The axis labels state the marker and fluorochrome used (e.g. CD4-FITC).

☒ The axis scales are clearly visible. Include numbers along axes only for bottom left plot of group (a 'group' is an analysis of identical markers).

☐ All plots are contour plots with outliers or pseudocolor plots.

☐ A numerical value for number of cells or percentage (with statistics) is provided.

Methodology

Sample preparation	KB31 cell line were stained with MN-Alexa647 as described in Material and Methods.
Instrument	The cell were analyzed using a FACSCanto II flow cytometer.
Software	Data was analyzed using the FlowJo v10.4.2 software.
Cell population abundance	N/A
Gating strategy	Only FSC/SSC gating was used. All negative and positive cells were showed on graph.

☐ Tick this box to confirm that a figure exemplifying the gating strategy is provided in the Supplementary Information.
